# KIFC1 is essential for acrosome formation and nuclear shaping during spermiogenesis in the lobster *Procambarus clarkii*

**DOI:** 10.18632/oncotarget.16429

**Published:** 2017-03-21

**Authors:** Dan-Dan Ma, Lian Bi, Wan-Xi Yang

**Affiliations:** ^1^ The Sperm Laboratory, College of Life Sciences, Zhejiang University, Hangzhou, Zhejiang, China

**Keywords:** procambarus clarkii, spermiogenesis, KIFC1, microtubule, RNAi

## Abstract

In order to study the function of kinesin-14 motor protein KIFC1 during spermatogenesis of *Procambarus clarkii*, the full length of *kifc1* was cloned from testes cDNA using Rapid-Amplification of cDNA Ends (RACE). The deduced KIFC1 protein sequence showed the highest similarity between *Procambarus clarkii* and *Eriocheir senensis* (similarity rate as 64%). According to the results of *in situ* hybridization (ISH), the *kifc1* mRNA was gathered in the acrosome location above nucleus in the mid- and late-stage spermatids. Immunofluorescence results were partly consistent with the ISH in middle spermatids, while in the late spermatids the KIFC1 was distributed around the nucleus which had large deformation and formed four to six nuclear arms. In the mature sperm, KIFC1 and microtubules were distributed around the sperm, playing a role in maintaining the sperm morphology and normal function. Overexpression of *P. clarkii kifc1* in GC1 cells for 24 hours resulted in disorganization of microtubules which changed the cell morphology from circular and spherical into fusiform. In addition, the overexpression also resulted in triple centrosomes during mitosis which eventually led to cell apoptosis. RNAi experiments showed that decreased KIFC1 protein levels resulted in total inhibition of spermatogenesis, with only mature sperm found in the RNAi-testis, implying an indispensable role of KIFC1 during *P. clarkii* spermiogenesis.

## INTRODUCTION

Kinesin is a superfamily of motor proteins that goes along the microtubule rails transporting cargoes. It often contains three domains, a conserved head including ATP-binding sequence and a microtubule-binding sequence, a curling stalk, and a variable globular tail. There are 14 families of kinesins according to the phylogenic results, among which only the kinesin-14 family contains C-terminal motor that moves from the minus end of microtubules to the plus end [1, 2]. The kinesin-14 family member KIFC1, as a transport machine for the cargoes, is usually found to be working with microtubules and play a part in spermiogenesis of a myriad of species.

Spermiogenesis refers to terminal phase of spermatogenesis and includes the transformation of the spermatids into mature and fertile sperms. There are various reports illuminating the different roles of KIFC1 plays in different species during spermiogenesis. Firstly, it takes part in the transformation of the acrosome. It is one of the components of the temporary structure acroframosome (AFS) that works as the scaffold for acrosome building in the Crustacea, Natantia such as *Expolaemon modestus* and *Macrobrachium nipponense* [3, 4]. It also takes part in the formation and maintenance of the *Eriocheir sinensis* acrosome structure [5]. Secondly, it participated in the nuclear morphogenesis. KIFC1 presents in the formation of manchette-like structure in the *Octopus tankahkeei*, presuming to constrain nucleus via mechanical force generated by its movement on the manchette microtubules [6]. For *Sepiella maindroni*, the expression of *kifc1* increased where and when the nuclei are changing [7]. It is also conjectured to participate in the formation of sperm head and tail in *Eumeces chinensis* [8].

There is no research of KIFC1 so far focusing on the Macrura, Crustacea, Reptantia. Red swamp lobster (*Procambarus clarkii*), the invasive species all around the world similar to the rusty crayfish (*Orconectes rusticus*) in the US [9] is a good example of Reptantia. On one side, such an invasive animal causes ecological damage to local species [10]. On the other side, the crayfish is becoming popular food in China for its palatable taste thus catalyzes the emergence of farms. Thus, the research of its reproduction is a guidance for controlling the population of the species.

In mammals, spermiogenesis is divided into four steps for the change of nuclei and acrosome [11]. As for *P. clarkii*, the most important changes during spermiogenesis include the formation of a modified nucleus with which to transmit genetic information and an acrosome presumably for effecting fertilization. Our experiment would focus on three questions: Firstly, Does Reptantia have the same structure of acroframosome as the Natantia? Secondly, is KIFC1 relative to the nuclei reshaping? Thirdly, does the KIFC1 participate in the formation of acrosome? In order to answer these questions, we cloned the full length of *kifc1* in *P. clarkii* in the testes and studied its expression pattern during the spermiogenesis using both the RNA probe as well as its antibody. Overexpression in cultured cells as well as RNAi were further used to clarify its functions during spermiogenesis. Our results will provide some evidence for the KIFC1 functions relating to the sperm maturation of crustaceans.

## RESULTS

### The spermiogenesis of *P. clarkii*

**Figure 1:**
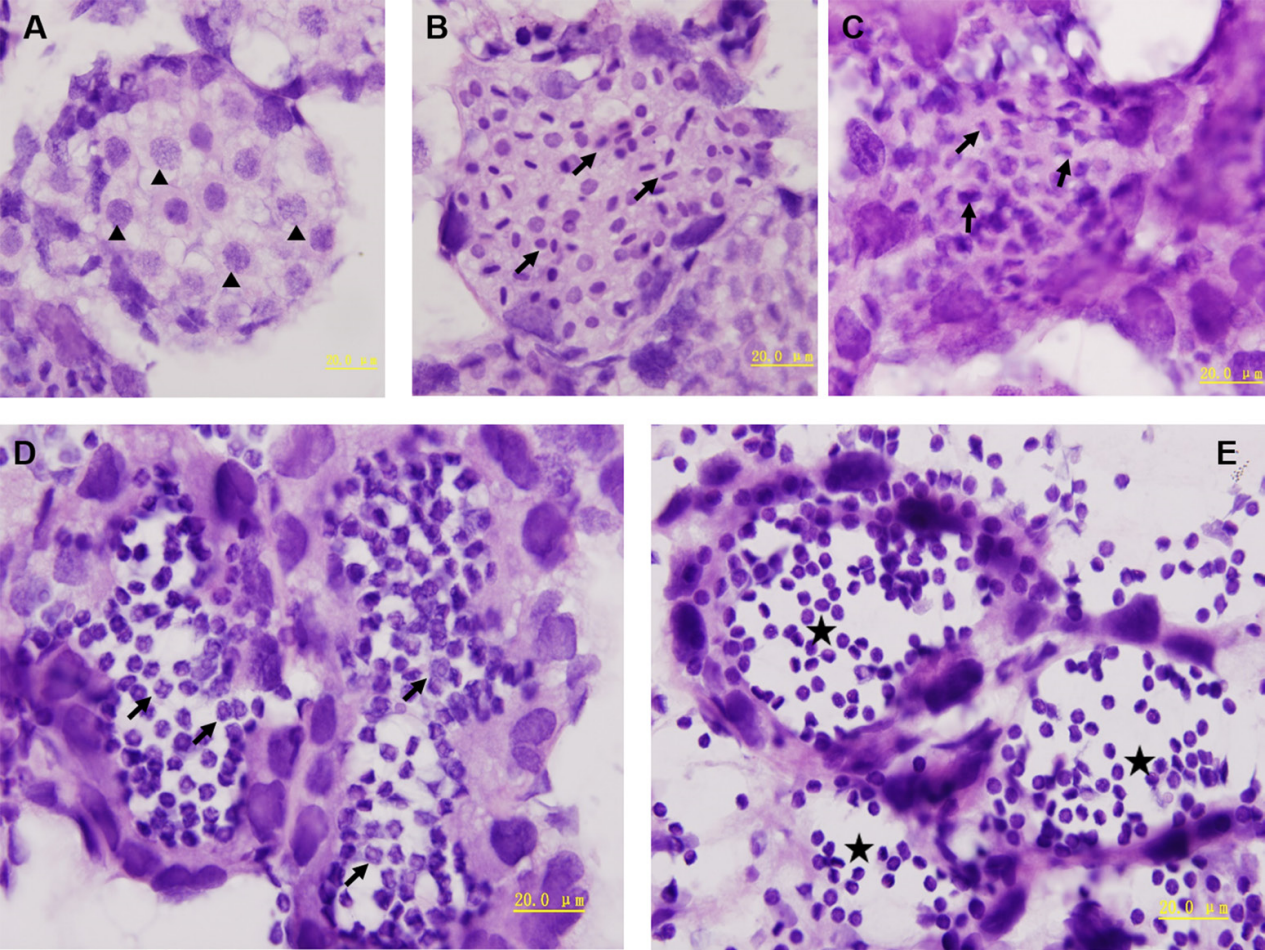
The spermiogenesis of *P. clarkii* The spermatocytes were the largest among spermatogenic cells, the nucleus was big and round (**A**, arrow heads). The spermatids were much smaller than spermatocytes (**B–D**). In the early-stage spermatids, the nucleus shrinks into a short rod-like shape and increases in density (B, arrows). In the mid-stage spermatids, the nucleus continues to shrink into bone-like shape (C, arrows). In the late-stage spermatids, the nucleus extends into four to six arms (D, arrows). The mature sperm is the smallest cells in testes that had a condensed nucleus with the arms wrapped around its body (**E**, stars).

There are three testes in *P. clarkii* that were located within the head cuirass, under the pericardial cavity and over the hepatopancreas. The testes were in ellipsoid or long capsule form. Inside the testes there existed different develop stages between senimiferous tubules. The spermiogenesis was divided into four stages according to the different spermatid morphology, which could be distinguished by their sizes and nuclear shapes (Figure 1). The spermatocyte has clear bounder of nucleus and cytoplasm, the chromatin was loosely distributed in the circular nucleus (Figure 1A). In early-stage spermatids the nucleus shrinks into a short rod-like shape and increases in density with small snacks of annulate lamellaes (Figure 1B). In the mid-satge spermatids the nucleus continues to change forming a bone-like shape with a large vesicle (pro-acrosome) lining proximal to the nucleus (Figure 1C). In the late-stage the spermatids are pro-mature, the nucleus extends into four to six arms with a deep invagination in the side of acrosome complex (Figure 1D). The mature sperm is a non-motile flattened spheroid that has a highly condensed nucleus with the arms wrapped around its body and a globate cup-shaped acrosome complex (Figure 1E).

### Full length of *P. clarkii kifc1*

**Figure 2:**
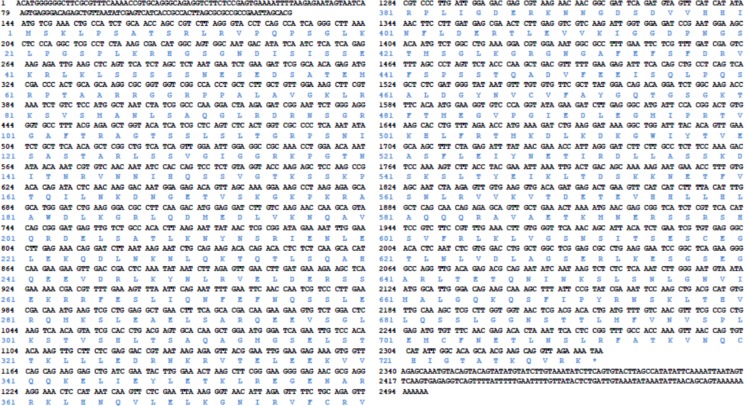
The full cDNA length of *P. clarkii kifc1 gene* The *kifc1* includes a 143 bp 5′ UTR, 160 bp 3′ UTR and a 2193 bp ORF encoding 731 amino acid.

The full length of *P. clarkii-kifc1* is 2499 bp in length (GenBank accession number: KM099199). It contains 143 bp 5′ untranslated region (UTR) and 160 bp 3′ UTR. The translated region is a 2193 bp open reading frame (ORF), encoding 731 amino acids of which the predicted molecular weight is 81 kDa (Figure 2, Supplementary Figure 1).

### The phylogenetic analysis of KIFC1 and putative protein analysis

**Figure 3:**
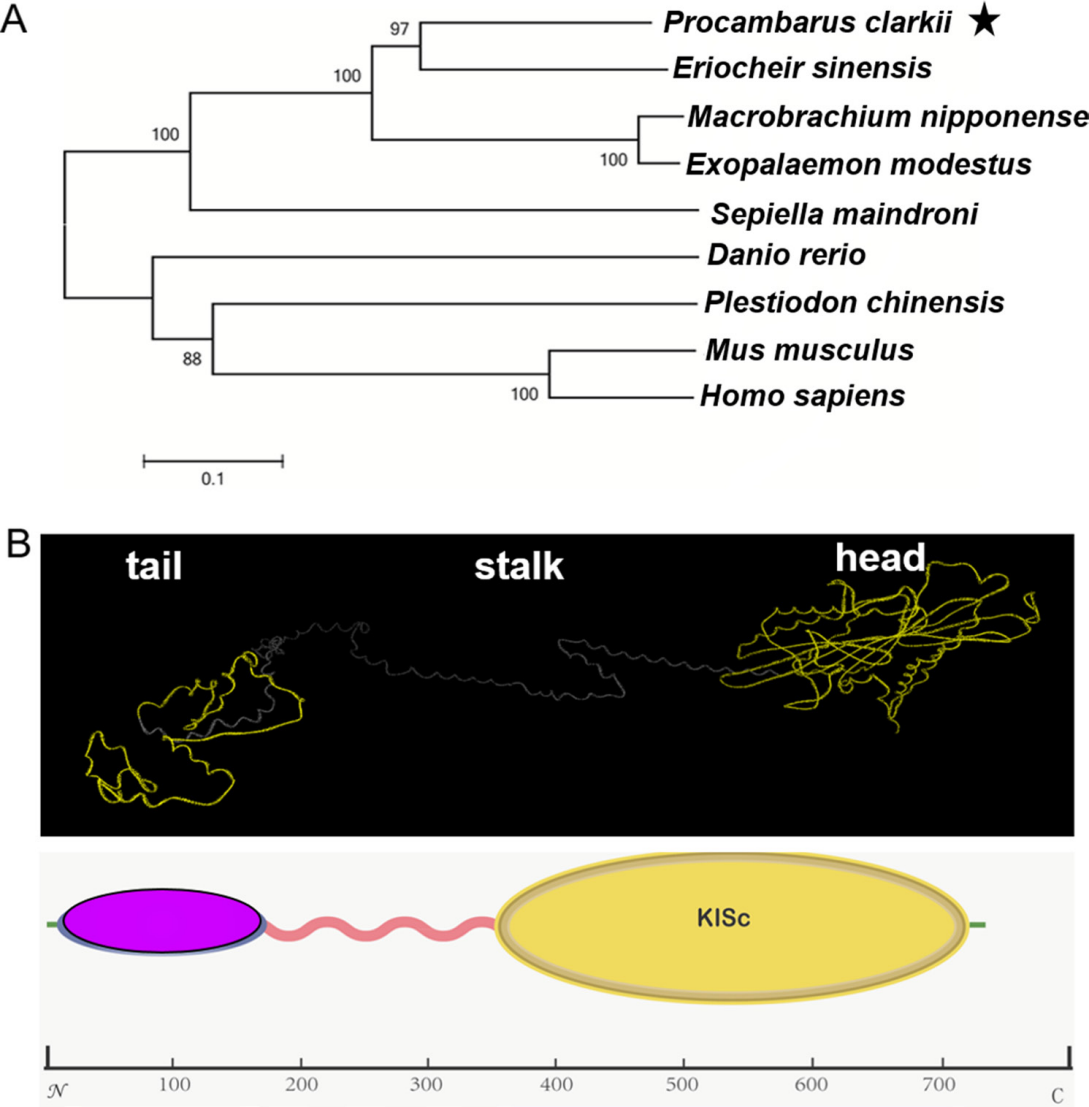
The phylogenetic analysis of KIFC1 and putative protein analysis Using the amino acid sequence of *P. clarkii*, the phylogenetic tree was analyzed using Mega 6.0, comparing KIFC1 homologues among various species including *Mus musculus* (uniprot: Q9QWT9), *Homo sapiens* (uniprot: Q9BW19), *Danio rerio* (uniprot: Q7ZZ74), *Plestiodon chinensis* (uniprot: A0A067XH81), *Sepiella maindroni* (uniprot: U5HTJ7), *Exopalaemon modestus* (uniprot: A0A088MIU8), *Macrobrachium nipponense* (uniprot: U5HTJ1), *Eriocheir sinensis* (uniprot: D9DBK9). The KIFC1 is more similar to its Reptantia cousin, *Eriocheir sinensis* (**A**). The C-terminus contains the conserved head (the yellow ball) that walks along the microtubules. The stalk region forms a helix region. The N-terminus contains the tail domain (the pink ball) that carries various cargoes, as predicted by I-TASSER (**B**).

Using the amino acid sequence of *P. clarkii*, the phylogenetic tree was analyzed by Mega 6.0. We compared *P. clarkii* KIFC1 sequence with that of *Mus musculus*, *Homo sapiens*, *Danio rerio*, *Plestiodon chinensis*, *Sepiella maindroni*, *Exopalaemon modestus*, *Macrobrachium nipponense*, *Eriocheir sinensis*. It was found that the KIFC1 is more similar to its Reptantia cousin, *Eriocheir sinensis*. The similarity rate was as high as 64% shown by NCBI blast results (Figure 3A).

We used I-TASSER to predict the three structural domains of KIFC1. The predict model of the KIFC1 protein consists of a typical C-terminal Kif head that walks along the microtubules, a N-terminus tail that carries specific cargoes and a helix stalk region that link the two domains (Figure 3B).

### The *kifc1* mRNA expression levels among *P. clarkii* tissues

**Figure 4:**
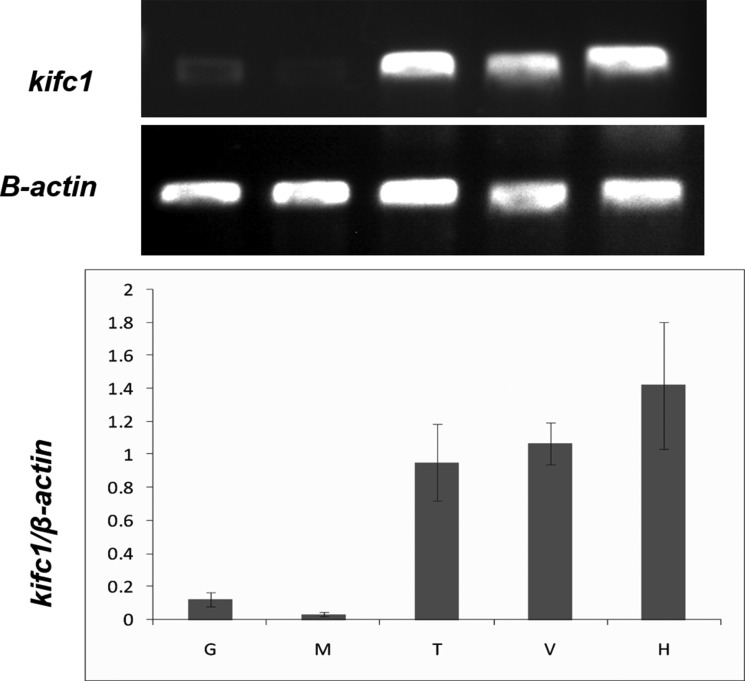
Semi-quantitative RT-PCR analysis of *kifc1* mRNA levels among different tissues β-actin was used as a positive control (lower panel). G. gills, M. muscle, T. testis, V. green glands, H. heart.

The analysis of relative content in Figure 4 demonstrated that *kifc1* is highly expressed in circulatory system (heart), excretory system (green gland), and reproductive system (testis). Very weak expression of *kifc1* was detected in the muscle and gill.

### The KIFC1 protein expression levels among P. clarkii tissues

**Figure 5:**
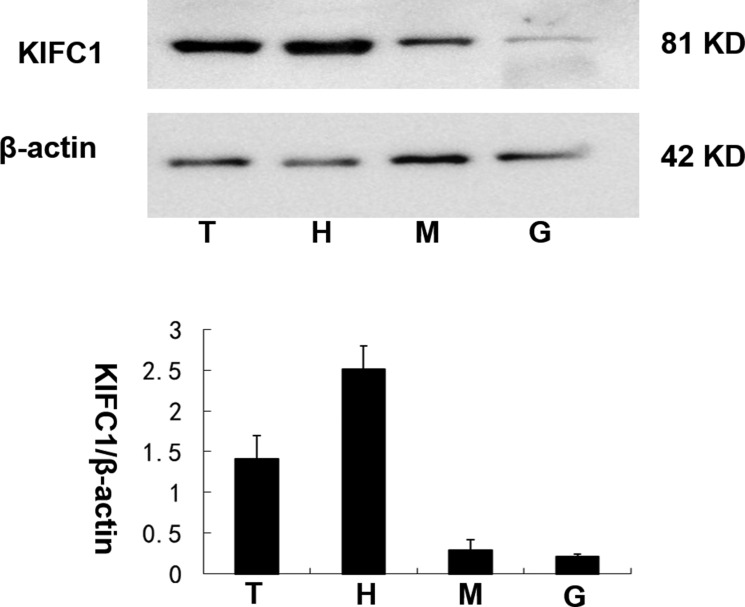
The KIFC1 protein expression levels among *P. clarkii* tissues KIFC1 was highly expressed in testis and heart while relatively low in muscle and green gland. β-actin was used as a positive control. T. testis, H. heart, M. muscle, G. green gland.

The *Exopalaemon modestus* polyclonal KIFC1 antibody was proved to be capable for *P. clarkii*-KIFC1 to get single, distinct and valid bands (Supplementary Figure 2). We compared the KIFC1 protein amount between testis and heart, muscle and green gland using β-actin as control. The expression of KIFC1 was higher in testis and heart than muscle and green gland (Figure 5).

### The *kifc1* mRNA expression pattern during *P. clarkii* spermiogenesis

**Figure 6:**
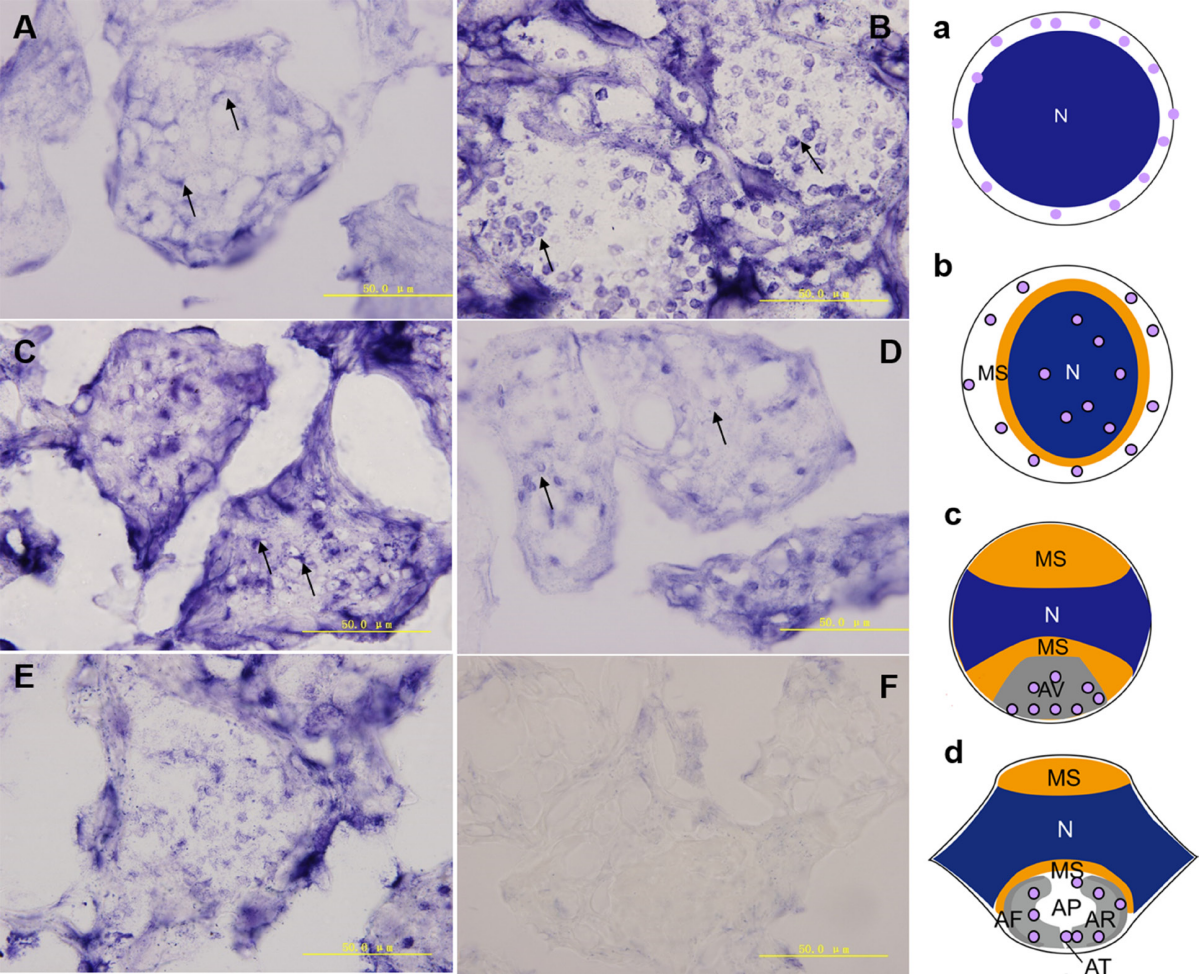
The *kifc1* mRNA expression pattern during *P. clarkii* spermiogenesis (**A–E**) In the spermatocytes, *kifc1* was randomly distributed in the cytoplasm (A, arrows). In early-stage spermatids, *kifc1* was highly transcribed and distributed in cytoplasm as well as nucleus (B, arrows). In the mid-stage spermatids, *kifc1* signals were gathered in the AV of acrosome location (C, arrows). In the late-stage spermatids, *kifc1* signals were gathered in the AF and AR of acrosome location (D, arrows). The *kifc1* mRNA was lowly expressed in mature sperm (E). The control slides incubated with sense *kifc1* probe showed no signal (**F**). N. nucleus, MS. membrane sheets, AV. acrosomal granule, AF. acrosomal filament, AR. acrosomal cap, AP. cloudy material in acrosomal cap, AT. acrosomal tubules. a-d. models of *kifc1* expression of A-D.

We used the anti-sense DIG-labelled *kifc1* probe to detect the *kifc1* mRNA spatial and temporal expression pattern in testes. In the spermatocytes, the *kifc1* was randomly distributed in the cytoplasm (Figure 6A, a), then the *kifc1* was highly transcribed and distributed both in nucleus and cytoplasm in early spermatids (Figure 6B, b). The *kifc1* signals was then gathered in the acrosome location above nucleus, rendering cargo transport along microtubule during acrosome formation in the mid-stage (Figure 6C, c) and late-stage spermatids (Figure 6D, d). With the reduced whole sperm gene transcriptional level as a result of highly condensed nuclear materials the *kifc1* mRNA was also low expressed in mature sperm (Figure 6E). The control slides incubated with sense *kifc1* probe showed no signal (Figure 6F).

### The KIFC1 protein expression pattern during *P. clarkii* spermiogenesis

**Figure 7:**
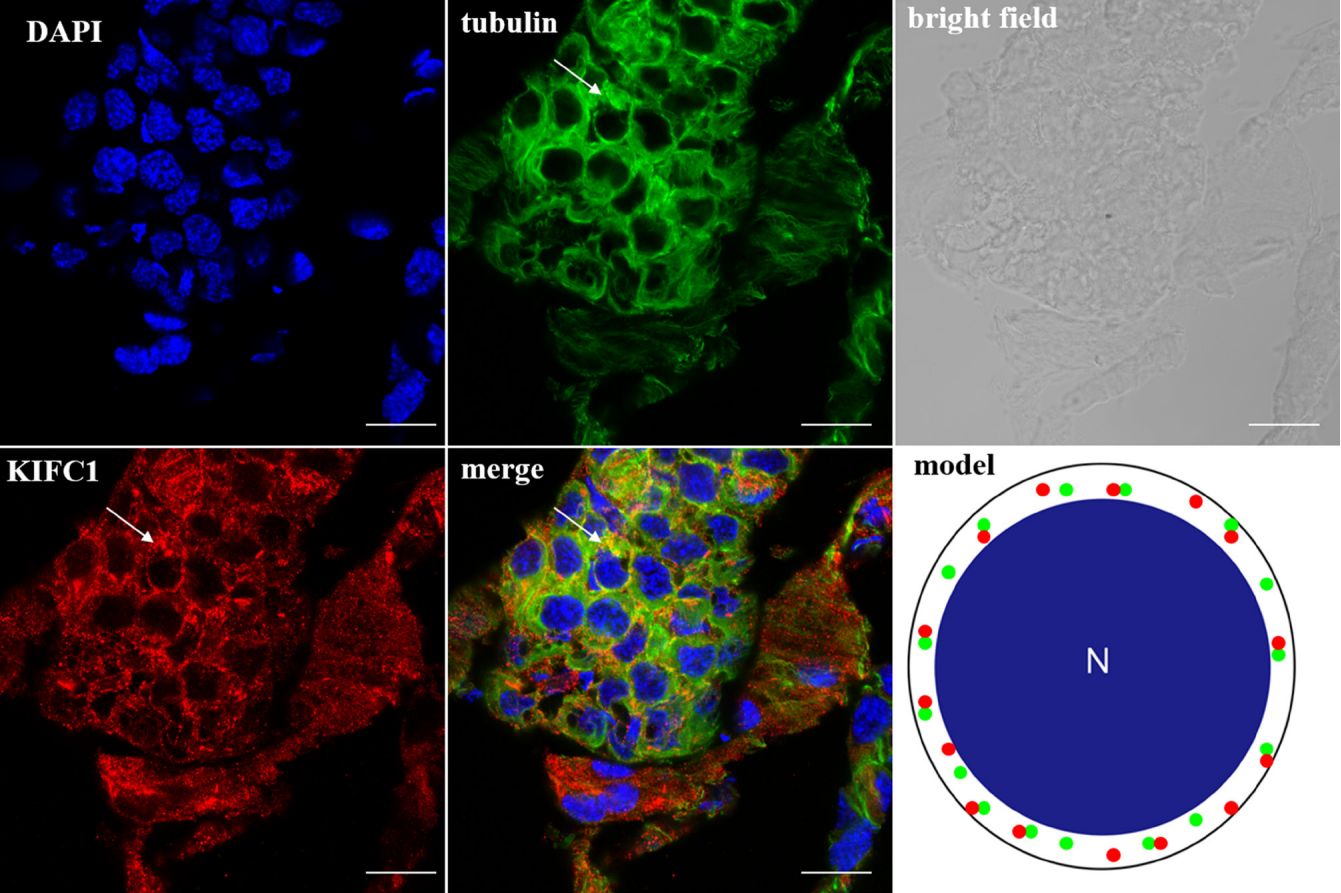
KIFC1 and microtubule expression pattern in spermatocytes KIFC1 and microtubules were randomly dispersed in the cytoplasm (arrows). Red: KIFC1. green: microtubules. blue: DAPI labeled nucleus. N. nucleus. (bar = 10 μm).

**Figure 8:**
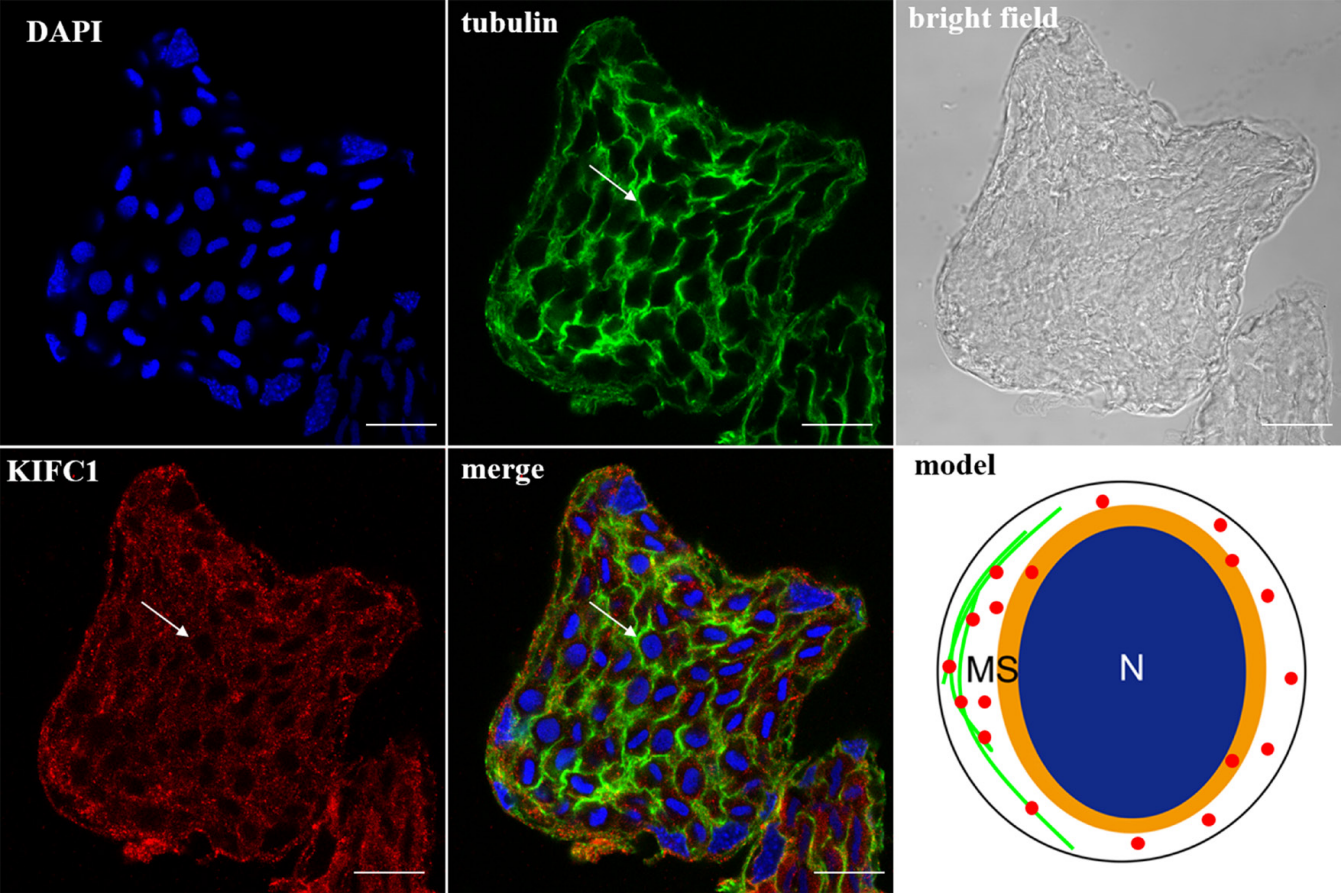
KIFC1 and microtubule expression pattern in early-stage spermatid The microtubules began to gather on one side of nucleus (tubulin, arrow). KIFC1 was dispersed in the cytoplasm (KIFC1, arrow). Red: KIFC1. green: microtubules. blue: DAPI labeled nucleus. N. nucleus, MS. membrane sheets. (bar = 10 μm)

**Figure 9:**
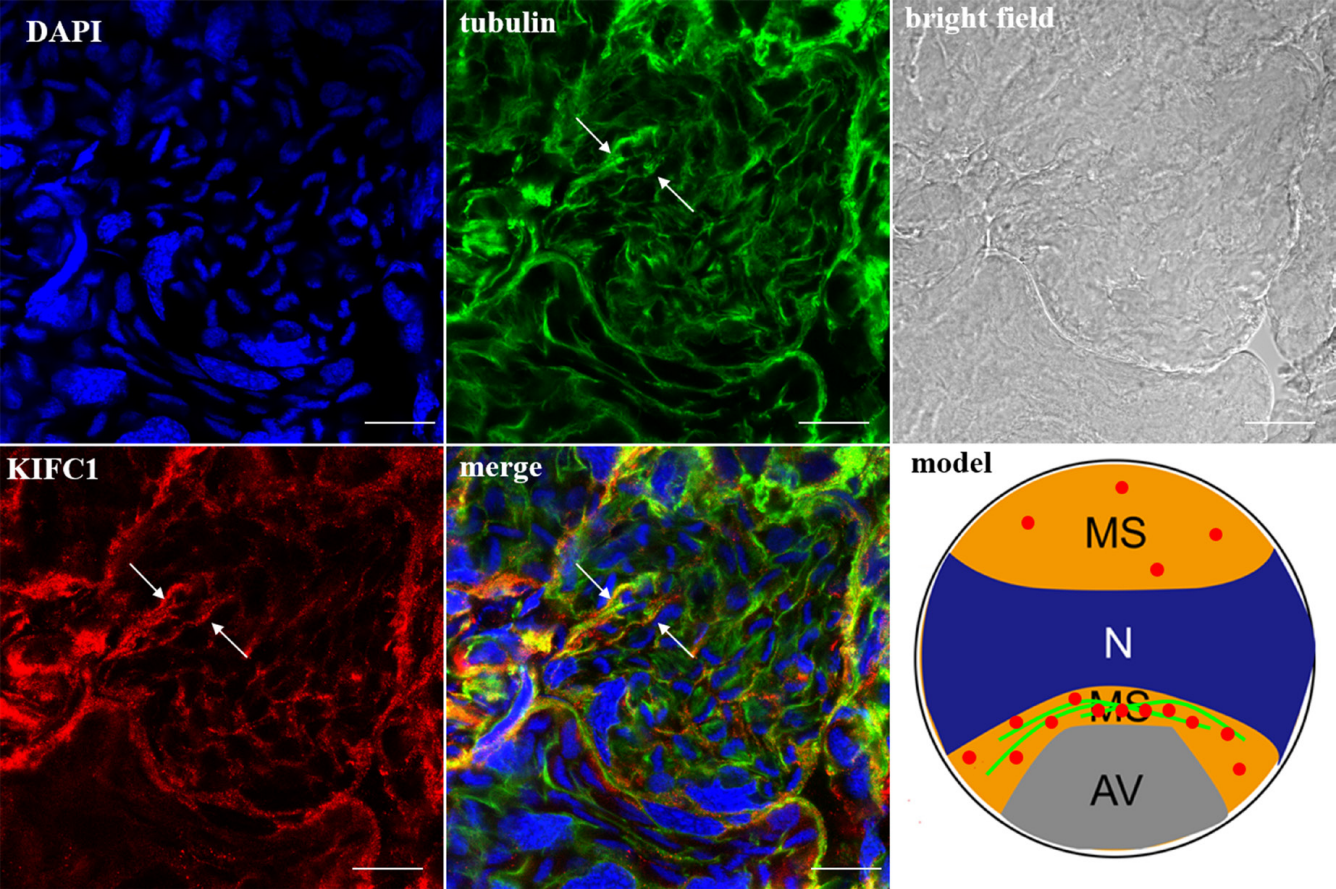
KIFC1 and microtubule expression pattern in mid-stage spermatids The microtubules and KIFC1 were co-localized at one side of the reshaping nucleus (merge, arrows). Red: KIFC1. green: microtubules. blue: DAPI labeled nucleus. N. nucleus, MS. membrane sheets, AV. acrosomal granule. (bar = 10 μm)

**Figure 10:**
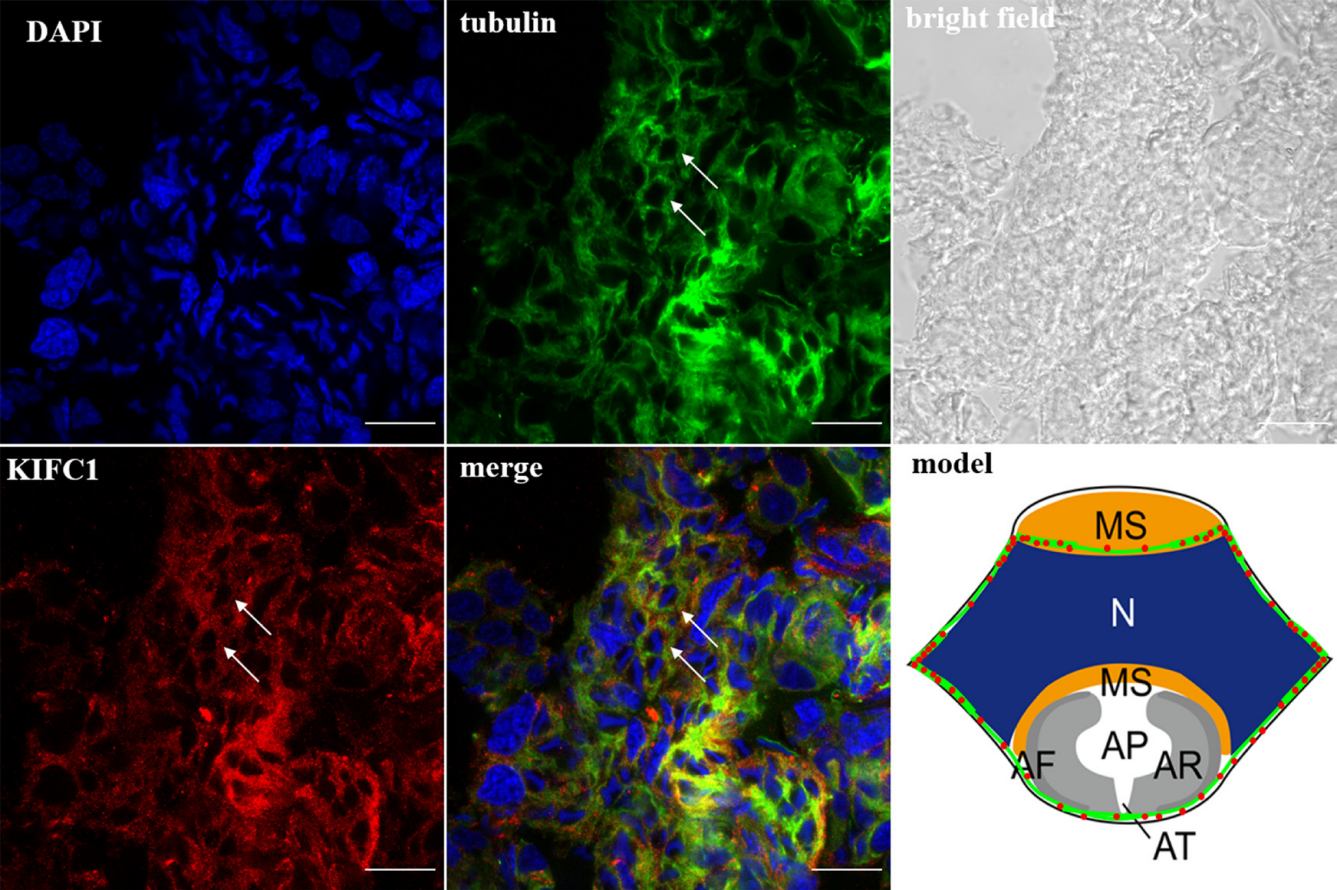
KIFC1 and microtubule expression pattern in late-stage spermatids KIFC1 and microtubules surrounded the reshaping nucleus of the late spermatids (arrows). Red: KIFC1. green: microtubules. blue: DAPI labeled nucleus. N. nucleus, MS. membrane sheets, AV. acrosomal granule, AF. acrosomal filament, AR. acrosomal cap, AP. cloudy material in acrosomal cap, AT. acrosomal tubules. (bar = 10 μm).

**Figure 11:**
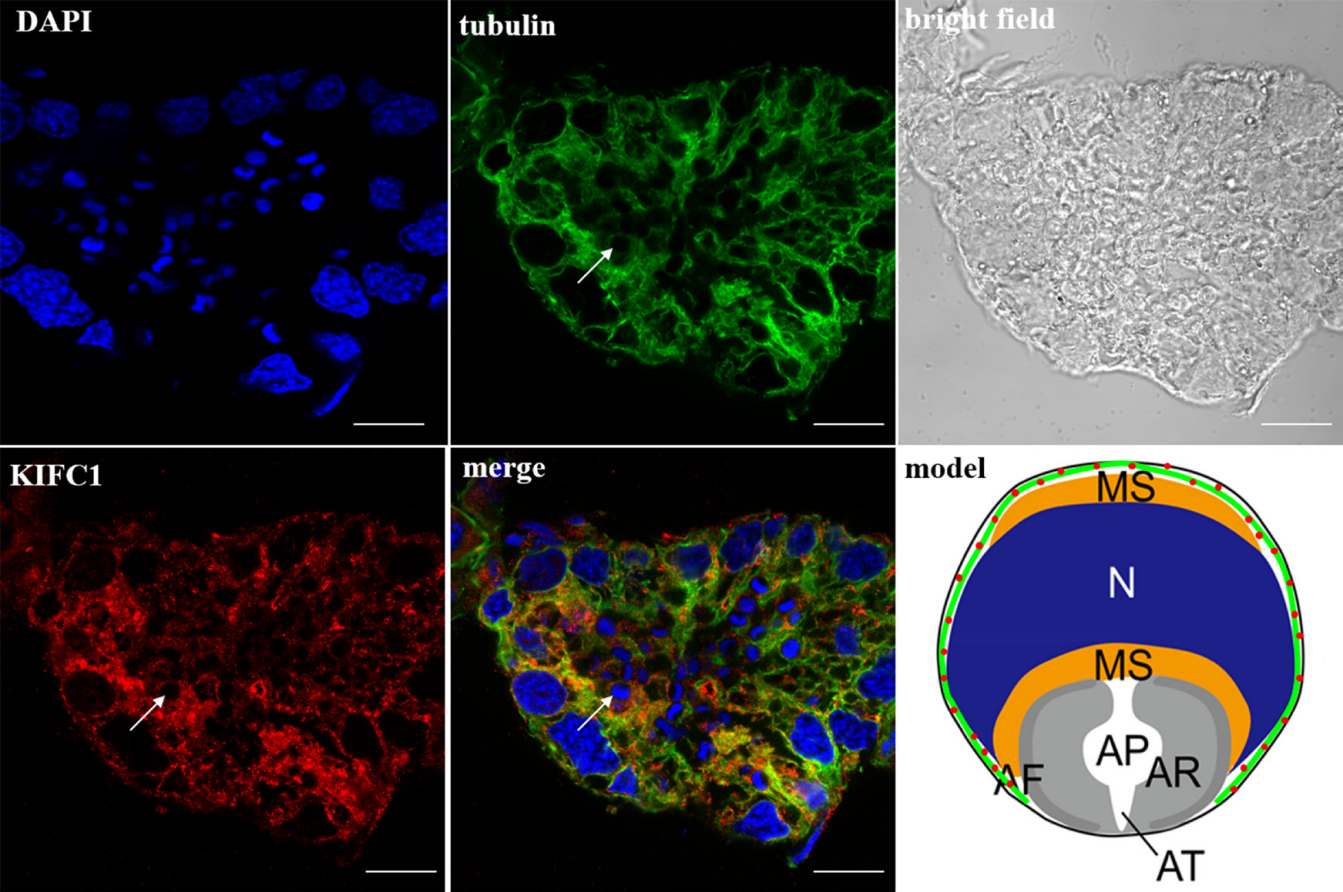
KIFC1 and microtubule expression pattern in mature sperm KIFC1 and microtubules were located around the whole mature sperm (arrows). Red: KIFC1. green: microtubules. blue: DAPI labeled nucleus. N. nucleus, MS. membrane sheets, AV. acrosomal granule, AF. acrosomal filament, AR. acrosomal cap, AP. cloudy material in acrosomal cap, AT. acrosomal tubules. (bar = 10 μm).

In the spermatocytes, the microtubules and KIFC1 were randomly dispersed and co-localized in the cytoplasm (Figure 7). In the early-stage spermatids, the microtubules started to gather in one side of the nucleus where the proacrosomal granules were formed, forming an umbrella-shaped structure (Figure 8), the KIFC1 signals were dispersed in the cytoplasm (Figure 8). In the mid-stage of spermiogenesis, the microtubules and KIFC1 were co-localized at one side of the reshaping nucleus of the mid-stage spermatids (Figure 9). During late spermiogenesis, the nucleus underwent dramatic morphological changes and formed 4-6 nuclear arms. It was found that KIFC1 as well as microtubules surrounded the changing nucleus of the late spermatids (Figure 10). KIFC1 and microtubules was located around the whole mature sperm and may be a support for the cellular structure (Figure 11).

### Effects of *P. clarkii* KIFC1 overexpression on GC1 cells

**Figure 12:**
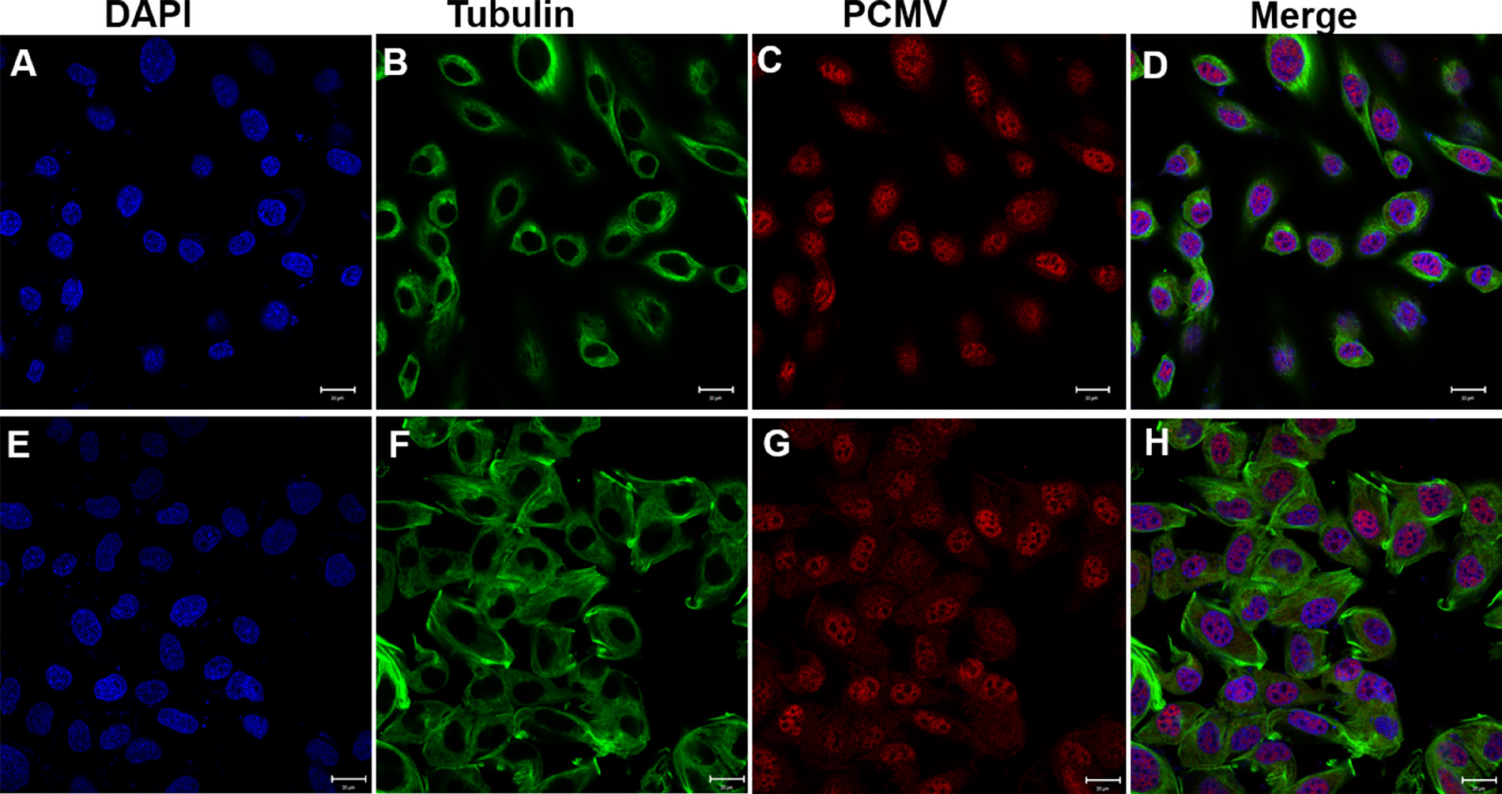
Effects of *P. clarkii kifc1* overexpression on GC1 cell morphology (**A**–**D**) control; (**E**–**H**) overexpression, A&E. nucleus, B&F. microtubule, C, pCMV, G, pCMV-KIFC1, D&H. merge. 24 hours after transfection, the GC1 cells of control groups were circular or oval, the microtubules were dispersed around the nucleus (D). In the experimental groups, the microtubules were irregularly arranged (F), the cell and nucleus shape changed from circular and oval to fusiform (E, H). (bar = 20 μm).

24 hours after transfection, the GC1 cells of control groups (transfected with pCMV-N-Flag) were circular or oval with microtubules dispersed around the nucleus (Figure 12D). However, in the experimental groups (transfected with pCMV-N-Flag-*P. clarkii kifc1*), the microtubules were irregularly arranged (Figure 12F) which eventually changed the cell and nuclear shape from circular and oval to fusiform (Figure 12E, 12H).

**Figure 13:**
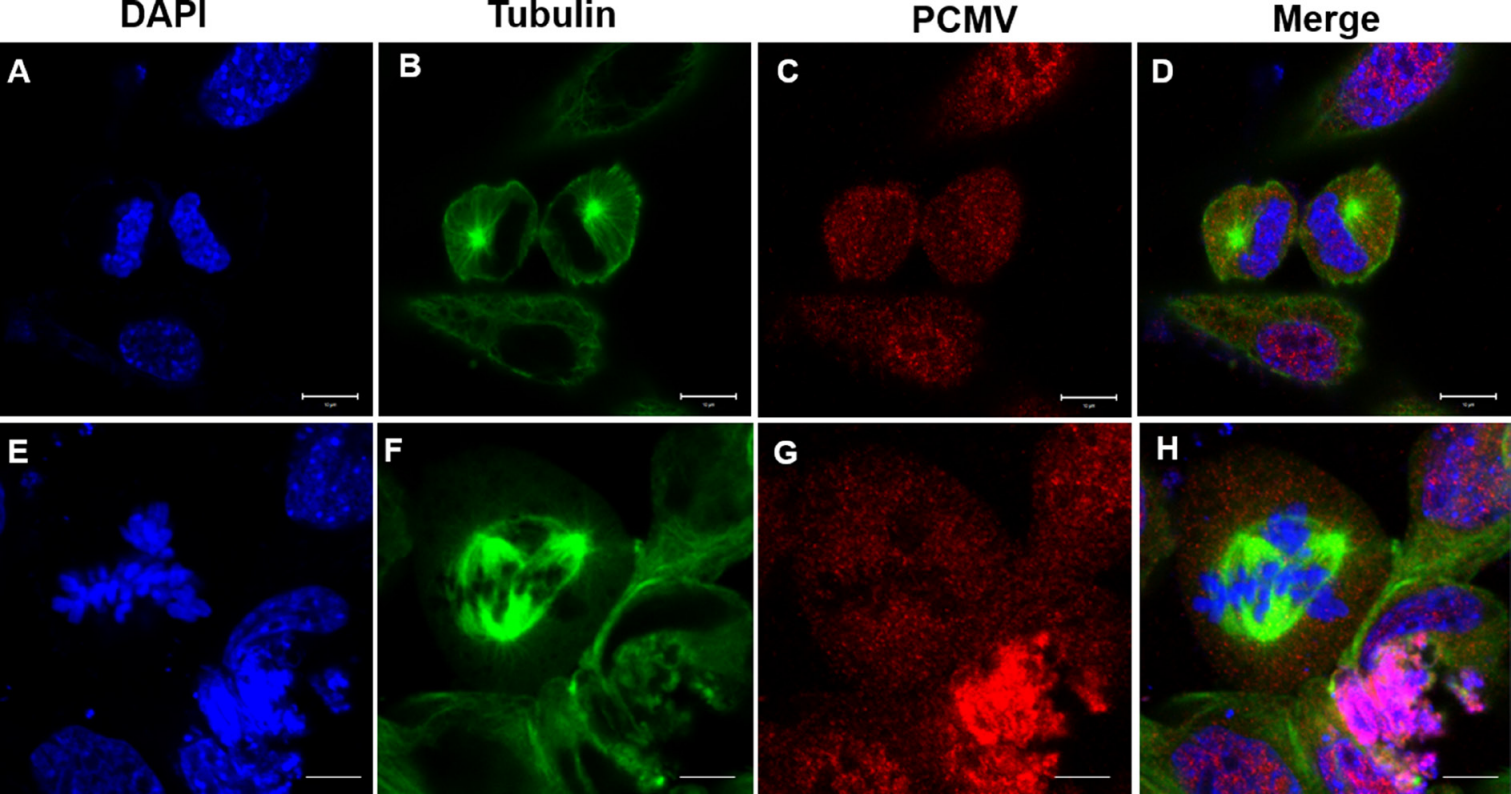
Effects of *P. clarkii kifc1* overexpression on GC1 cell mitosis (**A**–**D**) control; (**E**–**H**) overexpression, A&E. nucleus, B&F. microtubule, C, pCMV, G, pCMV-KIFC1, D&H. merge. The GC1 cells in control groups displayed normal mitosis with two spindle poles (B, D). In the experimental groups there existed abnormal mitosis with three spindle poles (F, H). (bar = 20 μm).

In addition to that, the GC1 cells in control groups displayed normal mitosis, which formed two spindle poles that separated the chromosome evenly into two daughter cells (Figure 13B, 13D). In the experimental groups there exist a portion of cells that exhibited abnormal mitosis, forming three spindle poles that separated the chromosome into three partitions and eventually drove the cells into apoptosis (Figure 13F, 13H).

### Effects of KIFC1 knockdown on *P. clarkii* testes

The efficiency of *kifc1*-siRNAs were examined *in vitro* using GC1 cells. It was found that all three siRNAs had a knockdown effect on the KIFC1 expression, the siRNA3 being the most effective siRNA which had a reduction of 81.4% over 60% of the samples (Supplementary Figure 3).

**Figure 14:**
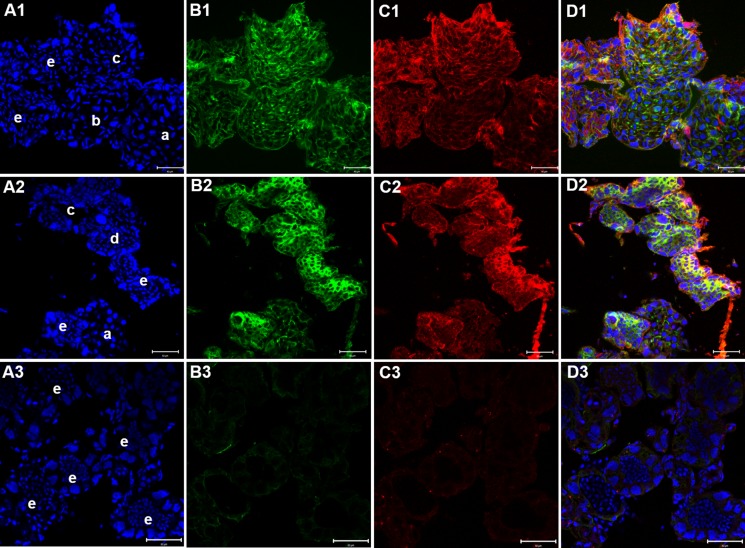
Effects of *kifc1* RNAi on *P. clarkii* testes (**A1–D1**) blank control; (**A2–D2**) negative control; (**A3-D3**) experimental groups. The testes of blank groups showed high expression of KIFC1 (C1) and microtubules (B1). Spermatocytes (A1a), various stages of spermatids (A1 b&c) and mature sperm (A1e) were observed in testes. The testes of negative control groups displayed similar pattern with the blank control (B1-B3). In the experimental groups, the expression levels of KIFC1 and microtubules were significantly reduced (C2, C3), spermatocytes and different stages of spermatids were not found, and only mature sperm were observed in the *kifc1*-siRNA groups (A3e). A1-C1, DAPI labeled nucleus; A2-C2, microtubules; A3-C3, KIFC1; A4-C4, merge. (bar = 50 μm).

The siRNA3 also had a knockdown effect of more than 70% in testes *in vivo*. 3 days after injection, the testes of blank groups (injected with PBS) showed high expression of KIFC1 (Figure 14C1) and microtubules (Figure 14B1) and normal morphology containing spermatocytes (Figure 14A1a), various stages of spermatids (Figure 14A1 b&c) and mature sperm (Figure 14 A1e), as showed by the nuclear shapes. High expression of KIFC1 and microtubules were detected in the testes of the negative and blank groups (Figure 14B1-B3). In the experimental groups, the sizes of testes were a bit smaller than the control groups, the expression levels of KIFC1 and microtubules were significantly reduced (Figure 14 C2&3), spermatocytes and different stages of spermatids were not found, and only mature sperm were observed in the *kifc1*-siRNA groups (Figure 14 A3e). After 9 days of injection, the amount of mature sperm was also significantly reduced in *kifc1*-siRNA groups (data not shown).

## DISCUSSION

Freshwater crayfish as the world's third largest crustacean species, is a significant worldwide aquatic food with a long history of human consumption [12]. Studies on *P. clarkii* mainly focused on toxicology [13, 14], ecology [15], neuron and behavior [16, 17], nevertheless their reproduction mechanism and regulation remain much unknown. Our previous work on *P. clarkii* investigated the mitochondrial prohibitin and its ubiquitination during crayfish spermiogenesis [18]. In this paper we studied the function of cytoskeletal microtubules and motor protein KIFC1 in the spermiogenesis of *P. clarkii*.

### Expression of KIFC1 in *P. clarkii* tissues

The kinesin 14 protein KIFC1, which is homologous to human HSET [19], *Xenopus* XCTK2 [20], *Drosophila* Ncd [21] and yeast Kar3 [22], is reported to be an ancient protein and played a role in various species. The three dimensional analysis of *P. clarkii* KIFC1 protein shows a 350-residue C-terminal globular motor domain that is conserved in the kinesin family [23], N-terminal species specific cargo binding domain that is slightly longer than that of other crustaceans, and a helical neck region that links these two domains. KIFC1 has been shown to participate in chromosomal and spindle movement during mitosis and meiosis, and transport membranous organelles and macromolecules fundamental to cellular functions [24]. It was found that *kifc1* mRNA was expressed in varieties of tissues in *P. clarkii* including testis, heart, green gland, muscle and gill, implying a wide function in *P. clarkii*, although there existed a difference in *kifc1* levels among tissues, with higher expressions in testis, heart, green gland while a relatively lower expressions in muscle and gill. The Western blot results were consistent with the semi-quantitative results and showed that KIFC1 protein was highly expressed in *P. clarkii* testes compared with other tissues. In this paper we focused on the KIFC1 function in spermiogenesis of *P. clarkii*, in order to shed a light on the reproduction mechanism and regulation.

### KIFC1 participates in *P. clarkii* acrosome biogenesis

According to Moses (1961) who observed the structural characterization of *P. clarkii* spermiogenesis by transmission electron microscope (TEM), the aflagellate sperm of *P. clarkii* can be homologized to flagellate forms only to the extent that both contain a modified nucleus with which to transmit genetic information and an acrosome presumably for effecting fertilization [25]. While the small snacks of annulate lamellaes formed during the middle spermatids which later turned into the PAF-positive acrosome complex, it drew our attention that how the protein and carbohydrate moieties move, arrange and organize into the functional and indispensable acrosome. It is clear that cells use a number of KIFs and tightly controls the direction, destination, and velocity of transport of various important functional molecules [26, 27], the C-terminal motor protein KIFC1 was supposed to have played a role in spermiogenesis of *P. clarkii*. The ISH results showed that the *kifc1* mRNA was primarily dispersed in the cytoplasm of early-stage spermatids, then gathered in the location where acrosome was formed in middle and late spermatids. The IF results also showed that, during acrosome formation the KIFC1 protein accumulated at one pole of the nucleus together with microtubules. Our previous studies on *E. modestus* [3] and *M. nipponense* [4] discovered that microtubules formed an umbrella-shaped structure over the nucleus (named acroframosome, AFS) during spermiogenesis, which was assumed to work as a scaffold for the kinesins and dyneins to transport cargoes that later were organized into functional acrosome. The kinesin superfamily motor proteins have been reported to move along microtubules carrying cargoes such as organelles, synaptic vesicles, protein complexes, mRNA, bare DNA, etc. [28, 29, 30]. KIFC1 as one member of the kinesin 14 family protein, was assumed to participate in the acrosome formation in spermiogenesis of *P. clarkii*.

### KIFC1 participates in *P. clarkii* nuclear shaping

The nuclear morphological changes are dominant in the evolution of sperm. In the mammals the sperm nuclear head was shaped by the microtubule-based manchette [31]. Kinesins like KIF27 and KIFC1-like motor were detected in the manchette, presumably promoting the nuclear shaping via the mechanical forces generated by walking on the manchette microtubules [7, 32]. Kinesins can not only move along microtubules, but also regulate the microtubule organization in different ways. For example, the kinesin-1 can change the microtubule orientation in the *Drosophila* axon via interplay with cortical dynein [33]. The kinesin-5 acts to restrain the number of minus end-distal microtubules that are transported into dendrites, suppression of kinesin-5 resulted in changes in dendritic morphology and microtubule organization [34]. During cell division, kinesin-12 (Kif15) and kinesin-5 (Eg5) cooperate to suppress microtubule depolymerization and drive the formation of parallel microtubule bundles that are essential for accurate chromosome segregation [35], kinesin-14 family proteins HSET/XCTK2 control spindle length by cross-linking and sliding microtubules [36]. In our present study it was observed that KIFC1 and microtubules were located around the reshaping nucleus in the late-stage spermatids, probably changing the nuclear shape in the traditional “manchette-like” way. As primitive culture of *P. clarkii* spermatogenic cells has not been tried out yet, and mammal cell lines have been proved to be a convenient way to testify protein functions of lower species like crustaceans [37], we also attempted to overexpress the *P. clarkii kifc1* in mouse spermatogonia germ cell line (GC1) to clarify its functions *in vitro*. It was found to induce the changes in microtubule organization as well as the cellular and nuclear morphologies of GC1 cells (Figure 12). Therefore, it is reasonable to assume that KIFC1 contributed in *P. clarkii* sperm nuclear shaping in the traditional microtubule-dependent manner.

### Functional analysis of KIFC1 in *P. clarkii* spermiogenesis

The full significance of this motor protein cannot be deduced from morphological observations alone. RNA interference (RNAi) is a biological process in which the exogenous or endogenous short double-stranded RNA molecules (dsRNA) inhibit gene expression or translation by neutralizing targeted mRNA molecules [38]. RNAi has been widely used to inhibit virus infections in shrimps [39, 40] and to study gene functions [41]. Whilst dsRNAs were mostly used in shrimps, small interfering RNAs (siRNAs) have also been proved to be capable of proficient gene-specific knockdown [42]. siRNAs combined into an nucleic acid complex, forming RNA induced silencing complex (RISC) which acted as a template to identify and destroy the mRNA of homologous sequence, resulting in specific inhibition of gene expression [43]. In the present study we designed and synthesized specific *kifc1*-siRNAs and screen out the effective ones to inhibit the KIFC1 expression in *P. clarkii*. It was found that the decreased levels of KIFC1 expression in testes were closely correlated with the abnormal testes in *kifc1*-siRNA groups, in which the spermiogenesis was totally inhibited when the KIFC1 expression was reduced, with the disappear of spermatocytes and spermatids, the mature sperm were also significantly reduced 9 days post injection. This is consistent with its homologous proteins in other species. For example, the decreased expression of KIFC1 in human testes was correlated with Globozoospermic defects [44]. KIFC1 has been reported to be essential for bipolar spindle formation and genomic stability in the primary human fibroblast IMR-90 cell, KIFC1 knock-down with specific shRNA induced 17% of cells with multiple microtubule organizing centers (MTOCs) in mitosis [45]. Overexpression of *P. clarkii kifc1* in GC1 cells also resulted in abnormal mitosis as reflected by appearance of three spindle poles (Figure 13). In one case, the loss of the kinesin-14 orthologues (KIN141, KIN142) in Tetrahymena led to severe defects in the chromosome segregation during both mitosis and meiosis [46], in another case the maize mutant *divergent spindle-1* (*dv1*) caused failures in meiotic spindle assembly and a decrease in pollen viability [47]. This may partly explain the abnormal spermiogenesis observed in *kifc1*-siRNA group.

In summary, the present study used the crayfish *P. clarkii* as a model to discuss the importance of kinesin-14 motor protein KIFC1 in the spermiogenesis of crustacean. It has been proved to participate in the sperm maturation in multiple ways, including acrosome formation and nuclear shaping, which are the prerequisites for sperm to fertilize an egg. Loss of KIFC1 resulted in total inhibition of spermiogenesis. It is speculated that the abnormal early spermatids underwent apoptosis as a result of unsuccessful meiosis, or that the developing spermatids underwent apoptosis as a result of incomplete structure in KIFC1-loss testes. We also attempt to overexpress the full length of *P. clarkii kifc1* in cultured cells to clarify its functions *in vitro*, and we hope this method could help crustacean specialists to explore gene functions in future studies.

## MATERIALS AND METHODS

### Animal preparation

The lobster (*P. clarkii*) was purchased from Luojia Village market (Hangzhou, China). The adult and active ones were selected for our experiment. We collect tissues to extract RNA and proteins including testes, hearts, muscles, and green glands from the males and ovaries from the females. For RNAi experiments, the adult and mature male red lobster with a consistent weight of 18 ± 2 g were used. When bought from the market, the lobster were acclimatized in the laboratory for more than 1 week (20°C, light/dark: 12 h/12 h) before experiments.

No specific permits were required for the collection of samples.

### Hematoxylin-eosin (HE) staining

The testes from adult male lobster were fixed in 4% paraformaldehyde for overnight and then dehydrated in a series of gradient alcohol as 70%, 80%, 95% for 15 min each and 100% twice for 15 mins. After that the samples were transferred into alcohol & xylene mixture for 15 min, and then in xylene for 10 min followed by 30 min in 50% xylene & 50% paraffin mixture. The samples were soaked in melting paraffin for 3 h before embedded in paraffin. When solidified the paraffin embedding fasts were cut into 6 μm sections. After rehydrated in gradient alcohol as 100%, 95%, 80%, 70% and water for 5 min each, the slides were stained with hematoxylin for 2 min and eosin-Y for 1 min. The slides were then dehydrated in gradient alcohol and permuted by xylene and sealed with neutral balsam. The pictures were taken by microscope (Olympus BX 40).

### RNA extraction and reverse transcript PCR

We used Phase Lock Gel^TM^ Heavy with Trizol A^+^ Reagent (Tiangen Biotech, Beijing, China) to extract total RNA. We collect the tissue of hearts, muscles, green glands, gills, and testis from lobster, treated with grinder in Trizol, then transferred to Phase Lock Gel^TM^ Heavy. With the treatment of chloroform, isopropanol and 75% ethanol, we centrifuge to get the RNA. The whole procedure is RNase-free.

For normal reverse transcription, we chose the PrimeScript^HRT^ Reagent Kit (Takara, Dalian, China). The 3′ and 5′ reverse transcription was processed following the protocol of Smart RACE cDNA Amplification Kit (CloneTech, Mountain View, USA).

### Full-length cDNA cloning

The primer used in this experiment was designed by Cong-cong Hou in the conservative region of *kifc1* of *M. rosenbergii* with the software Primer Premier 5. PCR programs were set as follows: 94°C for 4 min; 32 cycles of 94°C for 30 s, 60°C for 30 s, and 72°C for 30 s; and 72°C for 10 min for the final extension. The sample was separated by agarose electrophoresis. The purified DNA was ligated to PMD-18T and transformed to the competent cells, which were sent to Biosune Company (Shanghai, China) for sequencing.

Then with the sequence of the middle fragment, we designed nested primers for Rapid Amplification of cDNA Ends (RACE). Here are the PCR programs for the outer 3′ RACE: 94°C for 4 min; 6 cycles of 94°C for 30 s, 65°C (Touch- down -0.5°C) for 30 s, and 72°C for 40s; then 29 cycles of 94°C for 30 s, 62°C for 30 s, and 72°C for 40 s; 72°C for 10 min for the final extension. Then the production was put to inner extension by the same program except the extension temperature adjusted from 62°C to 61°C and one more cycle.

The programs of 5′ are as follow: 94°C for 4 min; 6 cycles of 94°C for 30 s, 70°C (Touch- down -0.5°C) for 30 s, and 72°C for 1 min; then 31 cycles of 94°C for 30 s, 64°C for 30 s, and 72°C for 1 min; 72°C for 10 min for the final extension.

Primers used are listed in the Table 1.

**Table 1:** Primers used in kifc1 cloning and semi-quantitative RT-PCR

Primer	Sequence	Function
F	CCCTTTTAAATCCCAAGCTGCTC	Intermediate segment
R	TTTGCTTACGGACAGACAGG	Intermediate segment
3′ F1	TGAAAGGACGTGGAAATGGC	3′ RACE
3′F2	TCTGAAGGATAAAGGCTGGATT	3′ RACE
5′R	TAAGTGTGGCAGACAACTCATCCCGC	5′ RACE
RT-actin-F	CCCAACAATGCTGACTGAA	Internal control of Semi-quantitative RT-PCR
RT-actin-R	CGGTGGTGGTGAAGGAATA	Internal control of Semi-quantitative RT-PCR
RT-kifc1-F	CCTGAAAGGACGTGGAAATG	Semi–quantitative RT-PCR
RT-kifc1-R	AAACGGAAGACGGAATGTGA	Semi-quantitative RT-PCR

### Putative protein sequence analysis and phylogenetic analysis

The putative KIFC1 protein sequence alignment and the phylogenetic tree were made by Mega 6. The phylogenetic tree were built by the Mega 6 using Neighbor-joining method and examined by bootstrap test with the replication of 1000. The protein 3-D structures were predicted by I-TASSER (http://zhanglab.ccmb.med.umich.edu/I-TASSER/) and the functional units in the proteins were analyzed in The Conserved Domain Database (http://www.ncbi.nlm.nih.gov/cdd), the similarities with their homologues were analyzed by using NCBI-blastp (https://blast.ncbi.nlm.nih.gov/Blast.cgi?PROGRAM=blastp&PAGE_TYPE=BlastSearch&LINK_LOC=blasthome).

### Semi-quantitative RT-PCR analysis of *kifc1* mRNA

We conducted Semi-quantitative RT-PCR to distinguish the *kifc1* mRNA expression of different tissues of lobsters. We used the reversed cDNA of heart, muscle, green gland, gill, and testis for the Semi-quantitative RT-PCR. We designed different pairs of primers of *kifc1* and control, β-actin. PCR programs were set as follows: 94°C for 4 min; 35 cycles of 94°C for 30 s, 60°C for 30 s, and 72°C for 30 s; 72°C for 10 min for the final extension. The products were examined by electrophoresis on 1% agarose gel. The bands were revealed by DNA gel green and analyzed by software Image J.

### Western blot

Four tissues including heart, muscle, green gland and testis were homogenized in RIPA Lysis Buffer (Beyotime Biotech, China) and the protease inhibitor PMSF (Beyotime Biotech, China). The samples were centrifuged in 13,000 rpm for 15 min at 4°C and the supernatants were collected. The total protein concentrations were measured and diluted to 1 μg/μL and conducted Western blot in 12% SDS-PAGE gels, each sample with a total protein amount of 10 μg. Transferred to PVDF membrane (Bio-Rad, California, USA), proteins were blocked in 5% non-fat milk for 2 hours. Then the membrane was incubated overnight in KIFC1 antibody (diluted 1:400 in PBS-milk) in 4°C and washed with PBST 3 times, 10 minutes each time. The rabbit KIFC1 polyclonal antibody used in this experiment was firstly designed for the KIFC1 of *Exopalaemon modestus* and described in the former study of our laboratory [3]. Membrane was then incubated in secondary antibody (HRP conjugated goat anti-rabbit IgG diluted in 1:1000 PBS-milk) for 1 hour. After washing 5 times (10 minutes each time), the image of the membrane was detected with enhanced chemiluminescent agents from SuperSignal West PicoTrial Kit (Thermo, Massachusetts, USA) by the chemiluminescence imaging. β-actin was used as control, the poly-anti-β-actin was bought from Sangon Biotech, China and used with 1: 1000 dilution in present study.

### *In situ* hybridization (ISH)

The ISH was conducted as described by Helmprobst *et al*. (2017) [48]. The testes were collected and fixed in 4% paraformaldehyde (PFA in PBS, pH 7.4) in the 4°C for 2 hours. After rinsing in PBS, the samples were dehydrated in 0.5 M sucrose (dissolved in PBS) in 4°C for another 2 hours, then embedded by Tissue-Tek O.C.T. Compound to store in the -40°C. For ISH, the samples were cut into 10 μm sections with Cryostat microtome. A length of 502 bp *kifc1* fragment was cloned from cDNA and ligated to PGEM-T Vector with Sp6 and T7 transcription sites. Sense and antisense probe was transcribed with Sp6 and T7 RNA transcription enzymes (Promega, Madison, Wisconsin, USA) and the NTP DIG RNA labeling Mix (Roche, Basel, Switzerland), according to the Manufacturer's protocols. After hybridization with the DIG-RNA antisense probes, the slides were incubated with Anti-Digoxigenin-AP Fab fragments (Roche, Basel, Switzerland) for overnight, then stained with NBT/BCIP mix (Promega, Madison, Wisconsin, USA). The slides were then dehydrated in gradient alcohol as 50%, 70%, 90%, and 100% for 15 min each and permuted by xylene before sealed with neutral balsam. The sense probes were used as negative control. The pictures were taken by microscope (Olympus BX40). The bands were analyzed by software Image J.

### Immunofluorescent assay (IF)

The testes were collected and fixed in 4% PFA in the 4°C overnight. After rinsing in PBS, the samples were dehydrated in 0.5 M sucrose in 4°C overnight then embedded by Tissue-Tek O.C.T. Compound to store in the -40°C. For IF, the samples were cut into 10 μm sections with Cryostat microtome. The sections were rehydrated with PBST (Triton) for 15 minutes, and then blocked with 5% BSA (PBST) for 1 hour in room temperature. Experiment groups were incubated with primary antibodies (anti-KIFC11:80 in BSA) and covered by parafilm at 4°C overnight. Slides of control group were incubated with BSA without adding primary antibody. After this, they were washed 3 times in 0.1% PBST for 15 min each. The tissue sections were then incubated at room temperature with the secondary antibodies (555 donkey-anti-rat IgG, 1:500, Alexa Fluor) and rat monoclonal anti-tubulin antibody (1:100 in BSA) (sigma, St. Louis, Mo., USA) for 1 hour. After that, the sections were washed 3 times in 0.1% PBST for 10 min each. Before mounting, the sections were added dropwise with DAPI (Beyotime, Dalian, China) to stain the DNA. Then with Antifade Mounting Medium (Beyotime Biotech, China), the sections were mounted and quickly observed with a Confocal Laser-scanning Microscope (CLSM 510) (Carl Zeiss, Germany).

### Overexpression of P. clarkii KIFC1 in GC1 cells

The *P. clarkii kifc1* expression construct was generated by inserting the full length *P. clarkii-kifc1* cDNA into pCMV-N-Flag (Beyotime Biotechnology, Shanghai, China) with BamHI/SpeI restriction sites, and verified by sequencing (Biosune Company, Shanghai, China). We then use EZNA Plasmid Miniprep 172 Kit II (Omega Bio-Tek, USA) to prepare the endotoxin-free plasmids, according to manufacturer's instructions. The mouse spermatogonia cell line GC1 was purchased from ATCC, and cultured in Dulbecco's Modified Eagle's Medium (DMEM) with penicillin (100 U/ml) and streptomycin (100 μg/ml) as well as 10 % heat inactivated fetal bovine serum (FBS). The cultures were incubated at 37°C with a humidified atmosphere of 5% CO2. The PCMV-N-Flag-*P. clarkii*-*kifc1* recombinant plasmids were then transfected into the 24-well plate GC1 cell cultures (with round cover glass slide inside each well) using Invitrogen^TM^ Lipofectamine® 175 3000 Kit, the pCMV-N-FLAG plasmids were used as a control. The overexpression was determined by IF staining 24 hours after transfection.

For IF, the cells were fixed with 4% PFA for 15 min and then wash with PBS, followed by permeabilization with 0.25% TritonX-100/PBS for 10 min. Block the slides with 1% BSA/PBST for 30 min, and then cells were incubated with rabbit monoclonal Flag antibody (1:100) overnight at 4°C. After this, the slides were washed with PBST (TritonX-100) and incubated with secondary antibodies for 1 hour at room temperature (Alexa Fluor 555-labeled Donkey Anti-Rabbit IgG (H+L), 1:500; Monoclonal Anti-alpha-Tubulin-FITC antibody, 1:100). After wash away the secondary, slides were incubated with DAPI (Beyotime, Shanghai, China) for 5min. Then slides were added dropwise by Antifade Mounting Medium (Beyotime, Shanghai, China) and sealed with nail polish. Observe and capture the pictures under the Confocal Laser-scanning Microscope (CLSM510; Carl Zeiss Germany).

### siRNA silencing

Three different sets of oligos were designed by Thermo Fisher BLOCK-iTTM RNAi Designer (http://rnaidesigner.thermofisher.com/rnaiexpress/) for knocking down of endogenous KIFC1, and one set of oligos were designed for GFP as negative control (Table 2). The siRNAs were synthesized using TaKaRa *in vitro* Transcription T7 Kit. Before injecting into lobster, the siRNA knockdown experiments were conducted *in vitro* via co-transfection of three different siRNAs with the PCMV-N-Flag-*P. clarkii-kifc1* recombinant plasmids into GC1 cells, 1 μg recombinant plasmid was co-added with 0.6 μg siRNA except the control groups. The knockdown efficiency was examined 48 hours after transfection by Western blot using PCMV antibody (1:1000).

**Table 2:** Sequences in siRNA synthesis

Name	Sequence
1-Oligo1	GATCACTAATACGACTCACTATAGGGCAGTCCTCTGTAGGTACCAAGAGCTTT
1-Oligo2	AAAGCTCTTGGTACCTACAGAGGACTGCCCTATAGTGAGTCGTATTAGTGATC
1-Oligo3	AACAGTCCTCTGTAGGTACCAAGAGCTCCCTATAGTGAGTCGTATTAGTGATC
1-Oligo4	GATCACTAATACGACTCACTATAGGGAGCTCTTGGTACCTACAGAGGACTGTT
2-Oligo1	GATCACTAATACGACTCACTATAGGGTGGAACTTGAGAAACAGGATCTTAATT
2-Oligo2	AATTAAGATCCTGTTTCTCAAGTTCCACCCTATAGTGAGTCGTATTAGTGATC
2-Oligo3	AATGGAACTTGAGAAACAGGATCTTAACCCTATAGTGAGTCGTATTAGTGAT
2-Oligo4	GATCACTAATACGACTCACTATAGGGTTAAGATCCTGTTTCTCAAGTTCCATT
3-Oligo1	GATCACTAATACGACTCACTATAGGGCAAGAAGAAGTTGACCGACTCAAAT TT
3-Oligo2	AAATTTGAGTCGGTCAACTTCTTCTTGCCCTATAGTGAGTCGTATTAGTGATC
3-Oligo3	AACAAGAAGAAGTTGACCGACTCAAATCCCTATAGTGAGTCGTATTAGTGATC
3-Oligo4	GATCACTAATACGACTCACTATAGGGATTTGAGTCGGTCAACTTCTTCTTGTT
GFP-1	GATCACTAATACGACTCACTATAGGGTCCTTCGCAAGACCCTTCCTCTATATT
GFP-2	AATATAGAGGAAGGGTCTTGCGAAGGACCCTATAGTGAGTCGTATTAGTGATC
GFP-3	AATCCTTCGCAAGACCCTTCCTCTATACCCTATAGTGAGTCGTATTAGTGATC
GFP-4	GATCACTAATACGACTCACTATAGGGTATAGAGGAAGGGTCTTGCGAAGGATT

The lobster were divided into three groups, each contained 20 individuals. The effective *kifc1-*siRNAs were then injected into the body cavity, each lobster was injected with 20 μg *kifc1*-siRNA (dissolved in PBS), the blank control groups were injected with PBS, and the negative control group were injected with same amount of *gfp*-siRNA. After 3 and 9 days the lobster were sacrificed and the testes were collected and fixed in 4% PFA in the 4°C refrigerator overnight. The samples were then incubated in 0.5 M sucrose in 4°C overnight, and then embedded by Tissue-Tek O.C.T. The efficiency of siRNA knockdown *in vivo* and the effects on testes were detected using IF staining. The experiments were conducted more than three times.

## SUPPLEMENTARY FIGURES



## Abbreviations

ISH: *in situ* hybridization
AFS: acroframosome
UTR: untranslated region
ORF: open reading frame
TEM: transmission election microscope
GC1: mouse spermatogonia germ cell line
RNAi: RNA interference
dsRNA: double-stranded RNA molecules
siRNAs: small interfering RNAs
MTOCs: multiple microtubule organizing centers
HE: Hematoxylin-eosin
RACE: Rapid Amplification of cDNA Ends
ISH: *in situ* hybridization
IF: Immunofluorescent
DMEM: Dulbecco's Modified Eagle's Medium
FBS: fetal bovine serum
PVDF: poly vinylidene flouride
RIPA: radio immunprecipitation assay
PMSF: phenylmethanesulfonyl fluoride
PBS: phosphate buffer saline
HRP: horseradish peroxidase
PFA: paraformaldehyde
GFP: green fluorescent protein
