# Supplementary material for: KIFC1 is essential for acrosome formation and nuclear shaping during spermiogenesis in the lobster Procambarus clarkii
Source: Oncotarget. 2017 Mar 21;8(22):36082–98. doi: 10.18632/oncotarget.16429 (PMC5482640; doi:10.18632/oncotarget.16429)
Supplement: Supplementary file 1 [file oncotarget-08-36082-s001.pdf]

KIFC1 is essential for acrosome formation and nuclear shaping during spermiogenesis in the lobster *Procambarus clarkii*

Supplementary Materials

1 ACATGGGGGGCTTCGCGTTTCAAAACCGTGCAGGGCAGAGGTCTTCTCCGAGTGAAAATTTTAAGAGAATAGTAATCA

79 AGTGAGGGACAGAGCTGTTAATATCGAGTCATCACCGCCACTTAGCGCCGCCGAATTAGCACG

144 ATG TCG AAA CTG CCA TCT GCA ACC AGC CGT CTT AGG GTA CCT CAG CCA TCA GGG CTT AAA

1 M S K L P S A T S R L R V P Q P S G L K

204 CTC CCA GGC TCG CCT CTA AAG CGA CAT GGC AGT GGC AAT GAC ATA TCA ATC TCA TCA GAG

21 L P G S P L K R H G S G N D I S I S S E

264 AAG AGA TTG AAG CTC AGT TCA TCT AGC TCT AAT GAA TCT GAA GAT TCG GCA ACA GAG ATG

41 K R L K L S S S S S N E S E D S A T E M

324 CGA CCC ACT GCA GCA AGG CGC GGT GGT CGG CCA CCT GCT CTT GCT GTT GGA AAG CTT CGT

61 R P T A A R R G G R P P A L A V G K L R

384 AAA TCT GTC TCC ATG GCT AAT CTA TCG GCC CAA GGA CTA AGA GAT CGG AAT TCT GGG AGG

81 K S V S M A N L S A Q G L R D R N S G R

444 GGT GCC TTT ACG AGA GCT GGT ACA TCA TCG CTC AGT CTC ACT GGT CGC CCC TCA AAT ATA

101 G A F T R A G T S S L S L T G R P S N I

504 TCT GCT TCA ACA GCT CGG CTG TCA TCA GTT GGA ATT GGA GGC CGC AAA CCT GGA ACA AAT

121 S A S T A R L S S V G I G G R K P G T N

564 ATA ACA AAT CGT GTC AAC AAT ATC CAC CAG TCC TCT GTA GGT ACC AAG AGC TCC AAG CCG

141 I T N R V N N I H Q S S V G T K S S K P

624 ACA CAG ATA CTC AAC AAG GAC AAT GGA GAG ACA GTT AGC AAA GGA AAG CCT AAG AGA GCA

161 T Q I L N K D N G E T V S K G K P K R A

684 GCA TGG GAT CTG AAG GGA CGC CTT CAA GAC ATG GAG GAT CTT GTC AAG AAC CAA GCA GTA

181 A W D L K G R L Q D M E D L V K N Q A V

744 CAG CGG GAT GAG TTG TCT GCC ACA CTT AAG AAT TAT AAC TCG CGG ATA GAA AAT TTG GAA

201 Q R D E L S A T L K N Y N S R I E N L E

804 CTT GAG AAA CAG GAT CTT AAT AAG AAT CTG CAG AAG ACA CAG ACA CTC TCT CAA GCA CAT

221 L E K Q D L N K N L Q K T Q T L S Q A H

864 CAA GAA GAA GTT GAC CGA CTC AAA TAT AAT CTT AGA GTT GAA CTT GAT GAA AGA AGC TCA

241 Q E E V D R L K Y N L R V E L D E R S S

924 GAA AAA CGA CGT TTT GAA AGT TTA ATT CAG AAT TTT GAA TTC AAC CAA TCG TCC CTT GAA

261 E K R R F E S L I Q N F E F N Q S S L E

984 CGA CAA ATG AAG TCG CTG GAG GCT GAA CTT TCA GCA CGA CAA GAA GAA GTG TCT GGA CTC

281 R Q M K S L E A E L S A R Q E E V S G L

1044 AAG TCA ACA GTA TCG CAC CTG ACG AGT GCA CAA GCT GGA ATG GGA TCA GAA TTG TCC ACA

301 K S T V S H L T S A Q A G M G S E L S T

1104 ACA AAG TTG CTT CTC GAG GAC CGT AAT AAG AGA GTT ACG GAA TTG GAA GAG AAA GTG GTT

321 T K L L L E D R N K R V T E L E E K V V

1164 CAG CAG AAG GAG CTG ATC GAA TAC TTG GAA ACT AAG CTT CGG GAA GGG GAG AAC GCG AGG

341 Q Q K E L I E Y L E T K L R E G E N A R

1224 AGG AAA CTC CAT AAT CAA GTT CTC GAA TTA AAG GGT AAC ATT AGA GTT TTC TGC AGA GTT

361 R K L H N Q V L E L K G N I R V F C R V

1284 CGT CCC TTG ATT GGA GAC GAG CGT AAG AAC AAC GGC GAT TCA GAT GTA GTT CAT CAT ATA

381 R P L I G D E R K N N G D S D V V H H I

1344 AAC TTC CTT GAT GAG CGA ACT CTT GAG GTC GTC AAG ATT GGT GGA GAT CCG AAT GGA AGC

401 N F L D E R T L E V V K I G G D P N G S

1404 ACA ATG TCT GGC CTG AAA GGA CGT GGA AAT GGC GCC TTT GAA TTC TCG TTT GAT CGA GTC

421 T M S G L K G R G N G A F E F S F D R V

1464 TTT AGC CCT AGT TCT ACC CAA GCT GAC GTT TTT GAA GAG ATT TCA CAG CTG CCT CAG TCA

441 F S P S S T Q A D V F E E I S Q L P Q S

1524 GCT CTC GAT GGG TAT AAT GTT TGT GTG TTC GCT TAT GGA CAG ACA GGA TCT GGC AAG ACC

461 A L D G Y N V C V F A Y G Q T G S G K T

1584 TTC ACA ATG GAA GGT GTC CCA GGT ATA GAA GAT CTT GAG GGC ATG ATT CCA CGG ACT GTG

481 F T M E G V P G I E D L E G M I P R T V

1644 AAG CAC CTG TTT AGA ACC ATG AAA GAT CTG AAG GAT AAA GGC TGG ATT TAC ACA GTT GAA

501 K H L F R T M K D L K D K G W I Y T V E

1704 GCA AGC TTT CTA GAG ATT TAT AAC GAA ACC ATT AGG GAT CTT CTT GCC TCT TCC AAA GAC

521 A S F L E I Y N E T I R D L L A S S K D

1764 TCC AAA AGT CTT ACC TAC GAA ATT AAA TTG ACT GAC AGC AAA AAG AAT GAA ACC TTT GTG

541 S K S L T Y E I K L T D S K K N E T F V

1824 AGC AAT CTA AGA GTT GTG AAG GTG ACA GAT GAG ACT GAA GTT CAT CAT CTT TTA CAT TTG

561 S N L R V V K V T D E T E V H H L L H L

1884 GCT CAG CAA CAG AGA GCA GTT GCT GAA ACT AAA ATG AAC GAG CGG TCA TCT CGT TCA CAT

581 A Q Q Q R A V A E T K M N E R S S R S H

1944 TCC GTC TTC CGT TTG AAA CTT GTG GGT TCA AAC AGC ATT ACA TCT GAA TCG TGT GAG GGC

601 S V F R L K L V G S N S I T S E S C E G

2004 ACA CTC AAT CTC GTG GAC CTG GCT GGC TCG GAG CGC CTG AAG GAA TCC GGC TCA GAA GGG

621 T L N L V D L A G S E R L K E S G S E G

2064 GCC AGG TTG ACA GAG ACG CAG AAT ATC AAT AAG TCT CTC TCA AAT CTT GGG AAT GTA ATA

641 A R L T E T Q N I N K S L S N L G N V I

2124 ATG GCA TTG GGA CAG AAG CAA AGC TTT ATT CCG TAT CGA AAT TCC AAG CTG ACG CAT GTG

661 M A L G Q K Q S F I P Y R N S K L T H V

2184 TTG CAA AGC TCG CTT GGT GGT AAC TCG ACG ACA CTG ATG TTT GTC AAC GTT TCG CCG CTG

681 L Q S S L G G N S T T L M F V N V S P L

2244 GAG ATG TGT TTC AAC GAG ACA CTA AAT TCA CTC CGG TTT GCC ACC AAA GTT AAC CAG TGT

701 E M C F N E T L N S L R F A T K V N Q C

2304 CAT ATT GGC ACA GCA ACG AAG CAG GTT AGA AAA TAA

721 H I G T A T K Q V R K *

2340 AGAGCAAATGTACAGTACAGTATATGTATCTTGTAAATATCTTCAGTGTACTTAGCCATATATTCAAAATTAATAGT

2417 TCAAGTGAGAGGTCAGTTTTATTTTTGAATTTTGTTATACTCTGATTGTAAATATAAATATTAACAGCAGTAAAAAA

2494 AAAAAA

Supplementary Figure 1: The full cDNA length of *P. clarkii kifc1* gene.

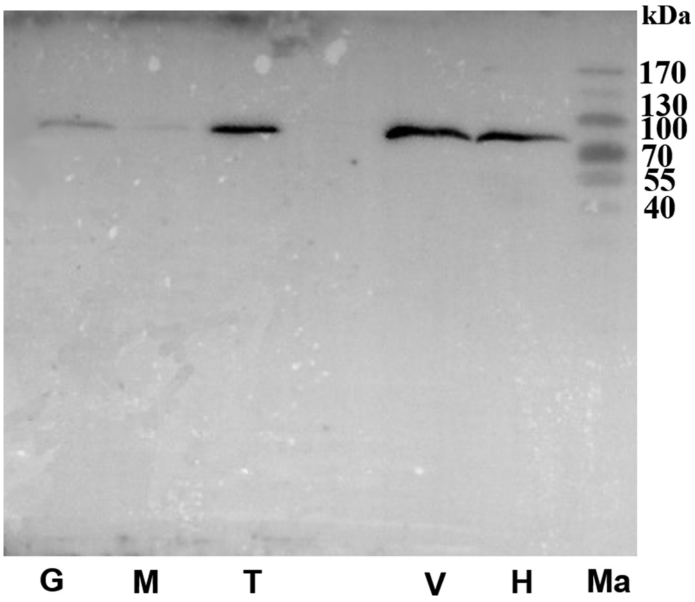

Supplementary Figure 2: The efficiency of KIFC1 antibody. The rabbit KIFC1 polyclonal antibody designed for *Exopalaemon modestus* was used in present study for *P. clarkii*. It showed single, distinct and valid bands for KIFC1 (81 kDa) among *P. clarkii* tissues. G. gills, M. muscle, T. testis, V. green glands, H. heart.

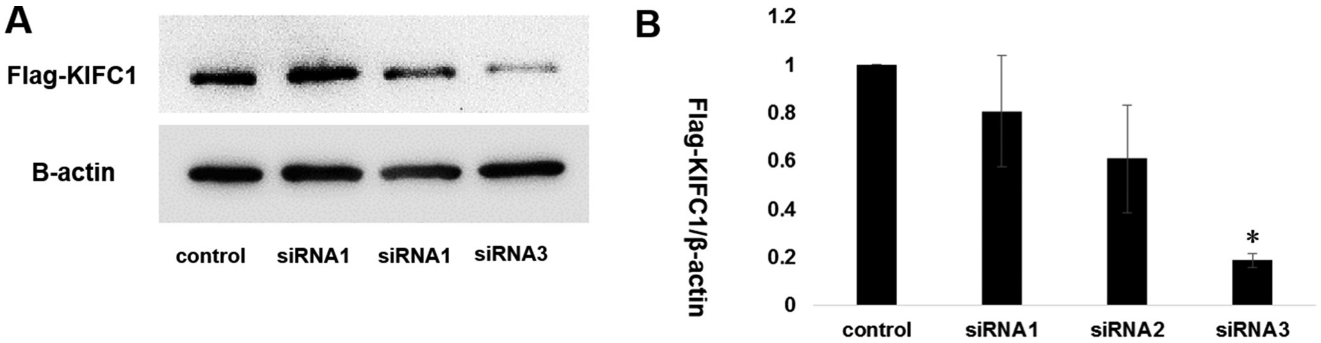

Supplementary Figure 3: The efficiency of *kifc1*-siRNAs in vitro using GC1 cells. It was found that all three siRNAs had a knockdown effect on the KIFC1 expression. The siRNA3 being the most effective siRNA which had a reduction of 81.4% in more than 60% of the samples.
